# *Thymus hirtus sp. algeriensis* Boiss. and Reut. volatile oil enhances TRAIL/Apo2L induced apoptosis and inhibits colon carcinogenesis through upregulation of death receptor pathway

**DOI:** 10.18632/aging.203552

**Published:** 2021-09-20

**Authors:** Fatma Guesmi, Sahdeo Prasad, Manel Ben Ali, Ismail A. Ismail, Ahmed Landoulsi

**Affiliations:** 1 Department of Experimental Therapeutics, The University of Texas MD Anderson Cancer Center, Houston, TX 77030, USA; 2 Laboratory of Biochemistry and Molecular Biology, University of Carthage, Faculty of Sciences of Bizerte, Zarzouna, Bizerte 7021, Tunisia; 3 Research and Development, Noble Pharma LLC, Menomonie, WI 54751, USA; 4 Department of Biology, College of Science, Taif University, Taif 21944, Saudi Arabia

**Keywords:** thymus hirtus Sp. algeriensis, HCT116, TRAIL, death receptors, colon cell carcinoma

## Abstract

Background: The aim of the study is to determine the anticancer activity of *Thymus algeriensis* (TS) and its underlying mechanisms using *in vitro* and in animal models.

Methods: HCT116 cells were treated with TS essential oil alone or with TRAIL, and then its anticancer effect was determined by using MTT assay, live dead assay, caspase activation and PARP cleavage. Further mechanisms of its anticancer effects was determined by analyzing expression of death receptor signaling pathway using Western blotting. A mouse model was also used to assess the antitumor potential of thyme essential oil.

Results: TS oily fraction showed tumor growth inhibitory effect even at lower concentration. TS induces apoptotic cell death as indicated by cleavage of PARP, and activation of the initiator and effector caspases (caspase-3, -8 and -9). Further, results showed that TS increases the expression of death receptors (DRs) and reduces the expression of TRAIL decoy receptors (DcRs). In addition, upregulation of signaling molecules of MAPK pathway (p38 kinase, ERK, JNK), down-regulation of c-FLIP, and overexpression of SP1 and CHOP were observed by TS. Further in animal model, intragastric administration of TS (12.5 mg/ml and 50 mg/ml) prevented colorectal carcinogenesis by blocking multi-steps in carcinoma.

Conclusion: Overall, these results indicate that thymus essential oil promotes apoptosis in HCT116 cells and impedes tumorigenesis in animal model. Moreover, thyme potentiates TRAIL-induced cell death through upregulation of DRs, CHOP and SP1 as well as downregulation of antiapoptotic proteins in HCT116 cells. However, therapeutic potential of TS needs to be further explored.

## INTRODUCTION

Cancer is still a major health issue with high care cost and causes physical and emotional difficulties for cancer patients. Besides preventive measures, several therapeutic modalities like surgery, chemotherapy and radiotherapy have been developed [1]. There is an unmet need for novel, affordable and effective anti-neoplastic medications [2]. With the development of new therapeutics, the incidence of colorectal cancer has decreased during the past two decades [3]. However, development of new drugs with higher efficacy and safety are still needed.

Apoptosis generally occurs via two major pathways, intrinsic and extrinsic. The extrinsic pathway of apoptosis in cancer cells takes place by binding of a ligand to the surface death receptor, while intrinsic pathway occurs through mitochondria [4]. TNF-related apoptosis inducing ligands (TRAIL) -bind to the death receptors. It has garnered much attention as a potential anticancer agent and improved effectiveness of therapeutic agents in combination [5]. Moreover, plant-derived secondary metabolites, known as phytochemicals, have also been shown to exert numerous beneficial effects on human health. Many preclinical studies have suggested anticancer functions of phytochemicals at the epigenetic and proteomic levels [6]. Natural compounds are known to target different signaling pathways involved in cancer progression, suggesting their potential to be successful anti-cancer agents [7]. Among various natural compounds, plants of *Lamiaceae* family have attracted much attention for their health benefits [8]. Many plants of *Lamiaceae* family have been investigated because of presence of high content of essential oils, which are widely used in pharmaceutical preparations, perfumery and cosmetics [9].

Among various plants of *Lamiaceae* family *Thymus* plants are rich in essential oil and contains oxygenated monoterpenes and monoterpene hydrocarbons as its major chemical components. Fifteen species (12 endemic) grow in northwest Africa, north of the Sahara desert (Morocco, Algeria, Tunisia, and Libya), with only three occur in the Iberian Peninsula [10]. In the traditional medicine, *Thymus hirtus* Willd. Ssp. *algeriensis* Boiss. and Reut. have been used as anti-inflammatory and anticancer agents and also as common tea and infusion in ethnophytotherapy [11]. The essential oil obtained from the aromatic parts of this plant has many phytotherapeutic effects and ontogenic features [12]. Thymus species has demonstrated significant free radical scavenging activity and proapoptotic effects in experimental models [13]. Thyme extract has also been shown to induce leukopoiesis and elevate thrombocyte count in blood [14]. Besides these, essential oil of Thymus species has antibacterial, antifungal and antiviral activities [15, 16].

We characterized here one of the aromatic and medicinal plants, *Thymus algeriensis*, that grows spontaneously in Tunisia. Previously, we and others have demonstrated the anticancer potential of *T. algeriensis* essential oil using *in vitro* models [11, 17]. However, the detail mechanism of its cancer preventive and therapeutic potential is still lacking. In this study, we report anticancer effects with mechanistic approach of essential oil from the aerial parts of *T. algeriensis* collected in the mountains of Orbata, Gafsa province of Tunisia. As of now, no reports on *in vivo* antitumor potential of TS have been described, although few studies have characterized the presence of essential oils in the plants. This plant is a rich composition of approximately 65 bioactive compounds, including viridiflorol, 2-carene, endo-borneol, terpinen-4-ol, camphor, eucalyptol, α-pinene and linalool, which varied from 34.38 to 42.48% of the total essential oil content [12]. To evaluate its possible use as an alternative or complementary cancer treatment, this report sheds light on the potent effect of thyme essential oil on colorectal cancer cells (CRC). This paper focuses on the TS essential oil-induced colon tumor cell apoptosis. We have unveiled the potent antitumor effect of TS, which is related to its ability to enhance TRAIL-induced apoptosis in human colon cancer cells (HCT116) by increasing TRAIL death receptors expression (DRs). Furthermore, *in vivo* study revealed the antitumor potential of *Thymus* species.

## RESULTS

### Terpenes profiling

**Figure 1:**
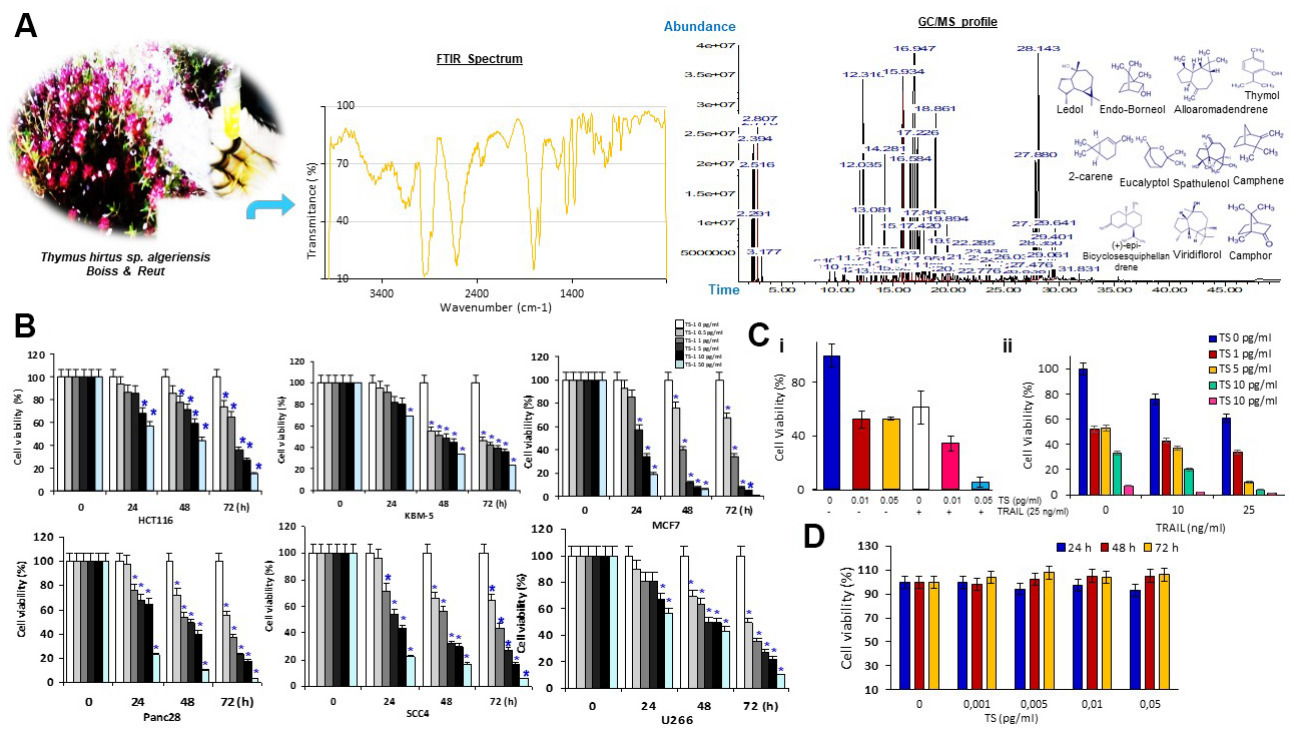
(**A**) Morphological features of TS plant samples at different phenological stages and structures of main components of the essential oil detected by GC/MS and FTIR. (**B**) Percentage of cancer cell viability after treatment with various concentrations of *Thymus hirtus Ssp. algeriensis* (TS) for different time points (0, 24, 48 and 72 hrs); * = statistical significance; *P˂*0.05. (**C**) TS sensitizes TRAIL-induced cytotoxicity. Briefly, HCT116 cells (5 × 10^3^ cells/well) were seeded in triplicate in 96-well plates. The cells were exposed to different concentrations of thyme volatile oil (0, 0.01 or 0.05 μl/ml) for 12 h, the medium was removed, and tumor cells were then incubated with 25 ng/ml TRAIL for additional 24 h. Cell viability was then determined by the MTT assay. (**D**) Effect of TS on RAW cell viability. RAW 264.7 cells, seeded in triplicate in 96-well plates, were exposed to different concentrations of TS (0, 0.001, 0.005, 0.01 or 0.05 μl/mL) and incubated for 24 h, 48 h and 72 h. Cell viability was analyzed with the MTT assay.

Oxygenated monoterpenoids and sesquiterpenoids are represented by high contents in thyme essence. Thymol, (+)-epi-bicyclosesquiphellandrene, ledol, camphor, linalool, 2-carene, terpinen-4-ol, endo-borneol, eucalyptol, alpha-pinene and alloaromadendrene are found to be predominated in *Thymus algeriensis*. Terpenic compounds of this species were quantified from different Tunisian mountainous area [18] and over phonological stages [12]. As seen in Figure 1A, the FT-IR spectral analyses of TS volatile oil indicated the presence of aromatic groups detected at a wave number of 1650 cm^-1^, carbohydrate compounds (3000 to 2800 cm^-1^), and aliphatic primary amine.

### TS and/or TRAIL induced cytotoxicity in cancer cells

To determine whether TS oil has anticancer activity, we performed cell viability assay. The cell viability of tumor cell lines was investigated by using MTT assay following 24, 48 and 72 hrs of treatment with TS oily fraction at 0.5–50 pg/mL. The results showed a decrease in cell growth with the increasing dose of TS (Figure 1B). Cancer cell lines responded to the antiproliferative effects of TS oil in a dose- and time-dependent manner. Incubation of cells with 50 pg/mL of essential oil significantly inhibited the growth of MCF7, Panc28 and SCC4 with a percentage of cell viability of 19% ± 0.54, 23% ± 2.08% and 22% ± 1.68% respectively after 24 hrs of treatment, 6% ± 0.22%, 10% ± 1.14% and 17% ± 0.48%, respectively after 48 hrs of treatment and 1% ± 0.08%, 3% ± 0.50% and 6% ± 0.02%, respectively after 72 hrs of treatment. TRAIL is new anticancer drug and it is still under clinical trials. However, some cancer cells display resistance against TRAIL. Therefore, we determined whether TS oil can enhance the anticancer effects of TRAIL. Cancer cells used in this report were sensitive to either TS or TRAIL alone. Moreover, TS essential oil significantly (*P* < 0.05) enhances TRAIL-induced human HCT116 cell growth inhibition in dose-dependent fashion (Figure 1C). As shown in Figure 1D, TS is not cytotoxic to RAW 264.7 macrophage cells.

### TS induces apoptosis in HCT116

**Figure 2:**
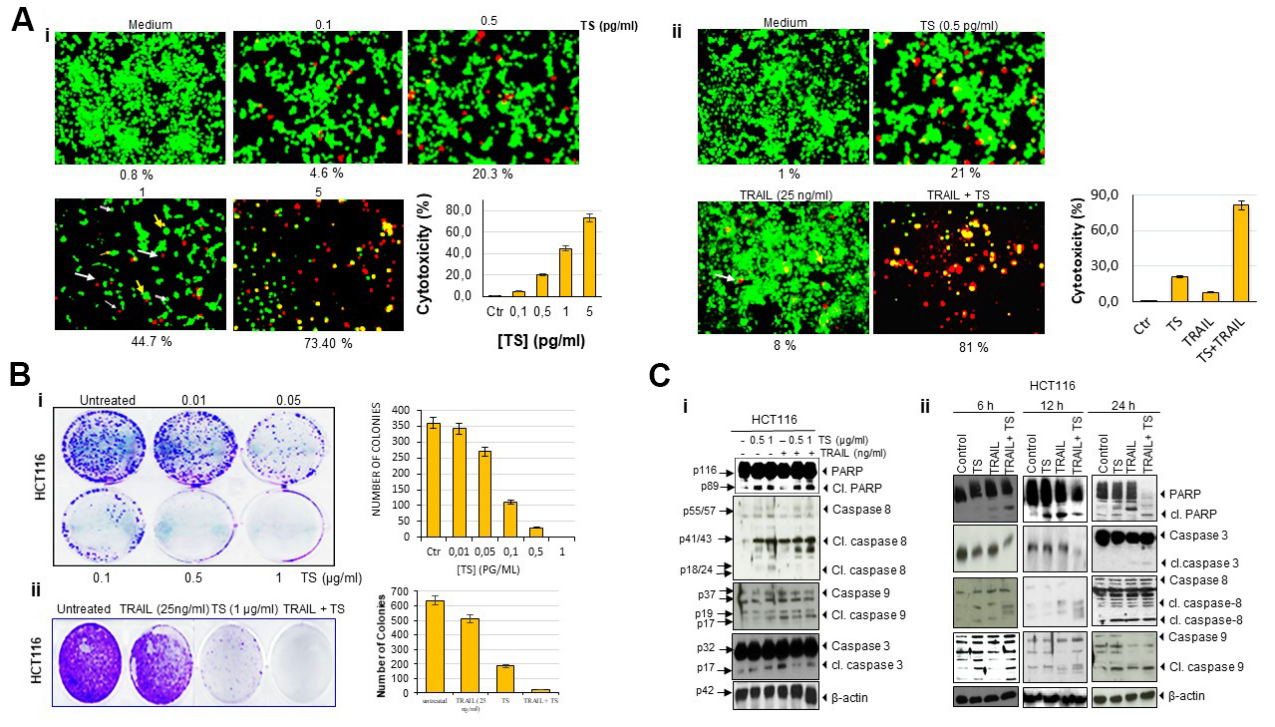
**Thyme volatile oil-sensitizes TRAIL induces colon cancer cell death.** (**A**) Human HCT116 cells (2 × 10^3^ per well) were treated with the indicated concentrations of TS for 24 h, stained with a Live/Dead assay reagent for 30 min and then analyzed for cell cytotoxicity under a fluorescence microscope. Values below each photomicrograph represent percentage of dead cells. Data represent the means of three independent experiments (i); colon cancer cells were exposed to 0.5 pg/ml thyme volatile oil for 12 h and rinsed with PBS. Cells were then treated with 25 ng/ml TRAIL for an additional 24 h. Cell death was analyzed by the LIVE/DEAD assay (ii). Orange arrows indicates necrotic cells; pink arrows indicates live cells and white arrows indicate apoptotic cell. (**B**) HCT116 (5 × 10^2^) cells seeded in 6 well plates were treated with different concentrations of TS (0-1 μg/ml), TRAIL (25 ng/ml) and TS + TRAIL for 9 days to form colonies and stained with clonogenic acid reagent to fix cells, and then incubated with crystal violet dye. Colony-forming ability was assessed by counting blue colonies. (**C**) TS sensitizes TRAIL-induced PARP cleavage and caspase activation in dose-dependent manner (i). Briefly, HCT116 cells (1 × 10^6^ per well) were pretreated with vehicle control (DMSO) or indicated doses of thyme essential oil for 12 h and then rinsed, TRAIL was then added for an additional 24 h. Whole-cell lysates were subjected to Western blotting analysis using relevant antibodies. TS sensitizes TRAIL-induced PARP cleavage and caspase activation in time-dependent manner. Colon cancer cells were exposed to thyme volatile oil and TRAIL at the indicated time points (ii). Whole-cell lysates were subjected to Western blotting analysis using relevant antibodies. Used blots were stripped and reprobed with β-actin antibodies to verify equal protein loading. These are representative results of three independent experiments.

Next, we confirmed the cytotoxic effects of TS by using live/dead assay. HCT116 cells were treated with different concentration of TS and analyzed under fluorescent microscope after staining with live/dead reagents. As seen in Figure 2Ai, TS volatile fractions-induced a dose-dependent cell death in HCT116 cells. In fact, thyme volatile oil dose-dependently increased the number of apoptotic cells from 1.6 to ~ 92.13% (*P*<0.05).

### TS potentiates TRAIL-induced cytotoxic effects in HCT116

As indicated in Figure 2Ai, we found that 0.5 pg/ml TS induce moderate cell death. Therefore, we investigated whether this dose of TS can enhance the apoptotic effect of TRAIL. We observed that 0.5 pg/ml TS and 25 ng/ml TRAIL alone moderately induced cell death. However, the combination of TS and TRAIL produced significantly enhanced cell death (81%) (Figure 2Aii). Thus, it is noteworthy that the combination of TS and TRAIL increased cytotoxicity.

### TS oily fractions reduced colony-forming ability of HCT116 cell lines

As growth of cancer cells in colony *in vitro* mimics the growth of tumor *in vivo*, we performed colony formation assay. Colony-forming assays measure the ability of cells in culture to grow and divide into groups. The present study was undertaken to investigate the effect of TS oily fraction on the colony-forming ability of colon cancer cell line. HCT116 cell line was seeded in 6-well plates and treated with TS oily fraction. After 9 days of incubation, we observed a large number of cancer cell colonies in control well. However, as seen in Figure 2Bi, the colony formation ability of cells was declined with increasing doses of TS. For dose-dependent investigation, HCT116 colonies were very sensitive to thyme essential oil and displayed dose dependent inhibition in colony formation. TS essential oil even at 0.5 and 1 μg/ml showed ~30% and 100% reduction in colony-forming ability respectively.

Further, we determined whether TS can increase the colony forming inhibitory effect of TRAIL. As shown in Figure 2Bii, treatment with TRAIL**/**Apo2L (25 ng/ml) alone reduced number of colonies whereas exposing colon carcinoma cells to TS and TRAIL in combination synergistically decreased the number of cancer cell colonies.

### TS potentiates the effect of TRAIL/Apo2L-mediated apoptosis through PARP cleavage and caspase cascades activation

We investigated whether TS enhances TRAIL**/**Apo2L-induced apoptosis in HCT116 cells. This was assessed by caspase activation and PARP cleavage. As shown in Figure 2Ci, pretreatment of colon cancer cells with either TS or TRAIL alone mediates significant cleavage of PARP and moderate caspase-3, -8 and -9 activation. However, when tumor cells were cotreated with 0.5 μg/ml or 1 μg/ml TS and 25 ng/ml TRAIL together, cleaved PARP was faintly enhanced and upstream and downstream caspases (casp. 3, 8, and 9) appeared to be activated (Figure 2Cii). Thus these findings suggest that TS enhances the anticancer effect of TRAIL.

### TS upregulates TRAIL receptors DR4 and DR5

**Figure 3:**
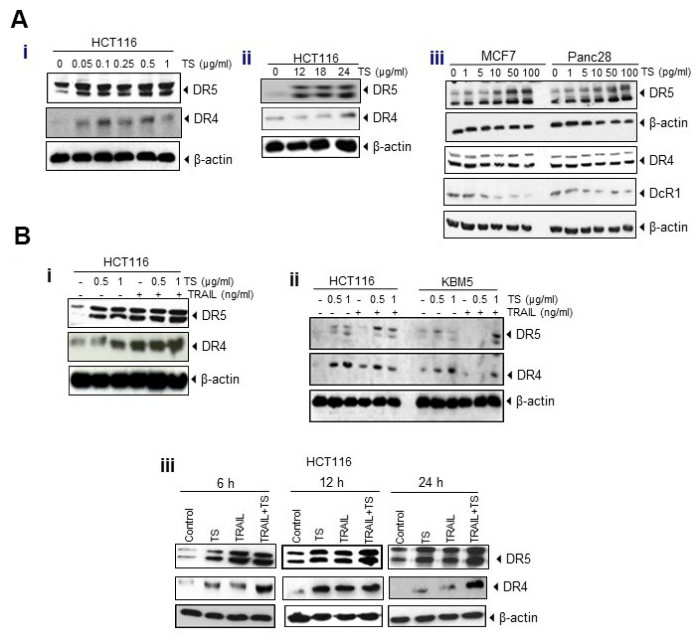
(**A**) Upregulation of DR4 and DR5 and reduction of decoy receptors (DcR1 and DcR2) by TS essential oil in dose (i)- and time (ii)-dependent manner in HCT116 and other cancer cells (iii). Tumor cells (1 × 10^6^ per well) were treated with various concentrations (A, left) of TS for 24 h or treated with 0.5 μg/ml TS for the indicated time points. Whole-cell extracts were prepared and subjected to Western blotting for DRs and DcRs. (**B**) TS enhances TRAIL-induced cell death through induction of DRs and down-regulation of DcRs expression in dose- (i) dependent manner and upregulation of death receptors in various time- (ii) periods in HCT116 and other tumor cells (iii).

This study is to underly the mechanisms of antitumor effects of oily fractions from *T. algeriensis*, in which HCT116 cells were treated with oily fraction of TS. The lysates were analyzed by Western blotting to examine the effect of TS on expression of death and decoy receptors. The results reveal that TS treatment induced the expression of death receptors in dose- (Figure 3Ai) and time- dependent (Figure 3Aii) manner. The effect of TS was more prominent on expression of DR5. Thus, treatment of human colon cancer cells with TS for 24 h appears to induce the expression of DR5 in MCF-7 and Panc28 cancer cells, indicating its non-specificity to cancer cells. In addition, TS suppressed the expression of decoy receptor DcR1. However, no effect was observed on the expression of DR4 and DcR2 in either MCF-7 or Panc28 cells (Figure 3Aiii).

TS is found to potentiate TRAIL-induced cell death through death receptors and inhibits growth and proliferation of colon cancer cells. Incubation of HCT116 with both TS and TRAIL increased the level of DR4/DR5 even more in dose- (Figure 3Bi, 3Bii) and time- (Figure 3Biii) dependent manner, compared with that in untreated cells.

### TS regulates Akt and MAPK pathways

**Figure 4:**
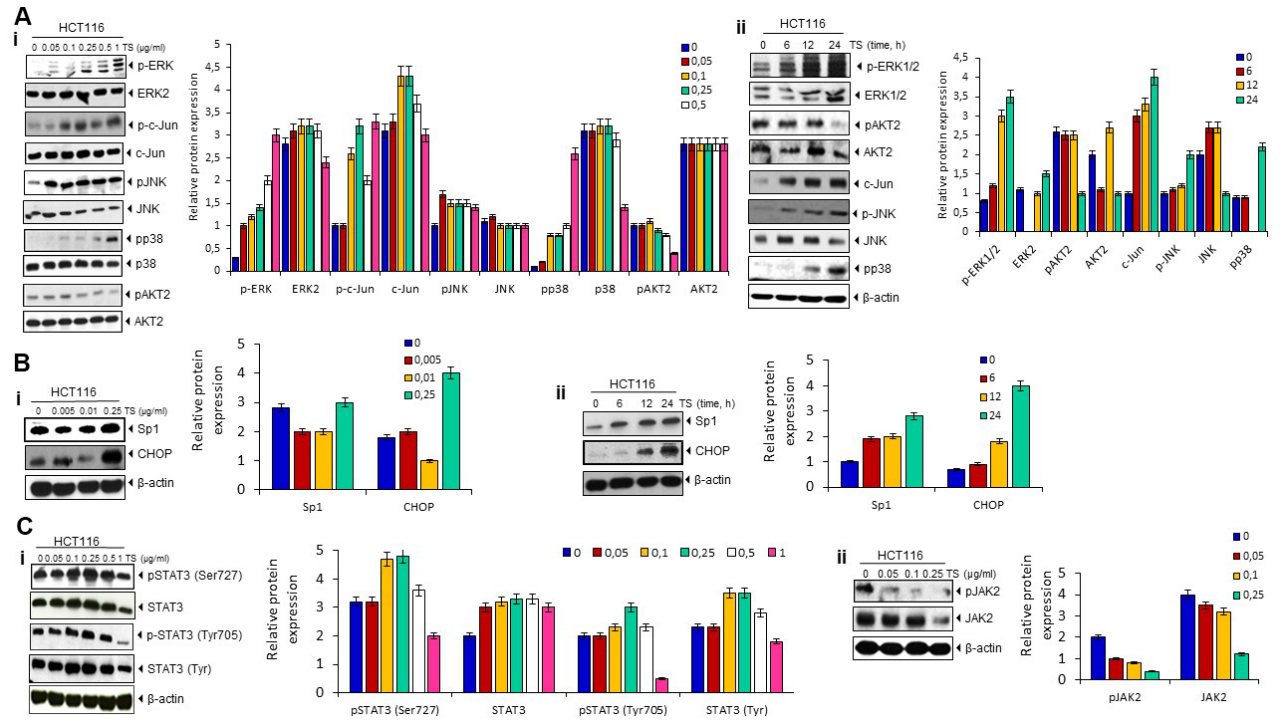
(**A**) Death receptor up-regulation is ERK1/2, p38 MAPK- and JNK-dependent. HCT116 cells (1 × 10^6^ cells/well) were treated with different dose of TS for 24 h or treated with 0.5 μg/ml TS for different time points. Whole-cell extracts were prepared and subjected to Western blotting for phosphorylated p38 MAPK, JNK, ERK1/2, c-jun, and Akt expression. The same blots were stripped and reprobed with p38, JNK, ERK1/2, c-jun, and Akt to verify equal loading. (**B**) TS mediates Sp1 and CHOP upregulation in human HCT116 cells in dose (i)- and time (ii)-dependent manner. HCT116 cells (1 × 10^6^ cells/well) were treated with different dose of TS (0-250 pg/ml) for 24 h or treated with 0.5 μg/ml TS for different time points. Whole-cell extracts were prepared and subjected to Western blotting for Sp1 and CHOP up-modulation and relative protein expression levels were quantified. The same blots were stripped and reprobed with β-actin to verify equal loading. (**C**) Thyme volatile oil inhibits STAT3 phosphorylation and JAK 2 levels in HCT-116 cell lines in a dose-dependent manner.

Because essential oils mediated apoptosis involves phosphorylation of MAPK [19], we investigated to determine whether TA also modulates MAPK pathway. We found that the treatment of TS in HCT116 cells induces the phosphorylation of ERK1/2, c-jun, JNK, and p38 MAPK in dose and time dependent manner. As MAPKs are known to responsible for upregulation of DR5 [20], induction of MAPK pathway by TS may contribute to the upregulation of DR5. However, TS decrease the phosphorylation of AKT2 in colon cancer cells (Figure 4A).

### TS up-regulates Sp1 and CHOP bZip transcription factors

Activation of MAPKs and other kinases (JNK) led to Sp1 translocation to the nucleus [21]. This Sp1 transcription factor is known to participate in cell surface expression of DR5 [22]. Therefore, we investigated whether TS can induce the Sp1 proteins. As shown in Figure 4B, treatment with TS (250 μg/ml) significantly induced the up-regulation of Sp1 and CHOP transcription factors in human colon cancer cell lines. This result indicates that Sp1 may be participating in TS-induced upregulation of DR5.

### TS suppresses STAT3 phosphorylation

STAT3 activity is known to regulate sensitivity to TRAIL-induced apoptosis in cancer cells [23]. Therefore, we examined whether TS can suppress the activation of STAT3. We observed that TS inhibited STAT3 (Tyr^705^) phosphorylation at higher dose but no suppression was observed at Ser^727^ of STAT3 (Figure 4Ci). Moreover, suppression in upstream kinase JAK was observed at higher dose of TS in colon cancer cells (Figure 4Cii).

### Both thyme essential oil and TRAIL/Apo2L represses expression of multiple gene products in colon carcinoma cell lines

**Figure 5:**
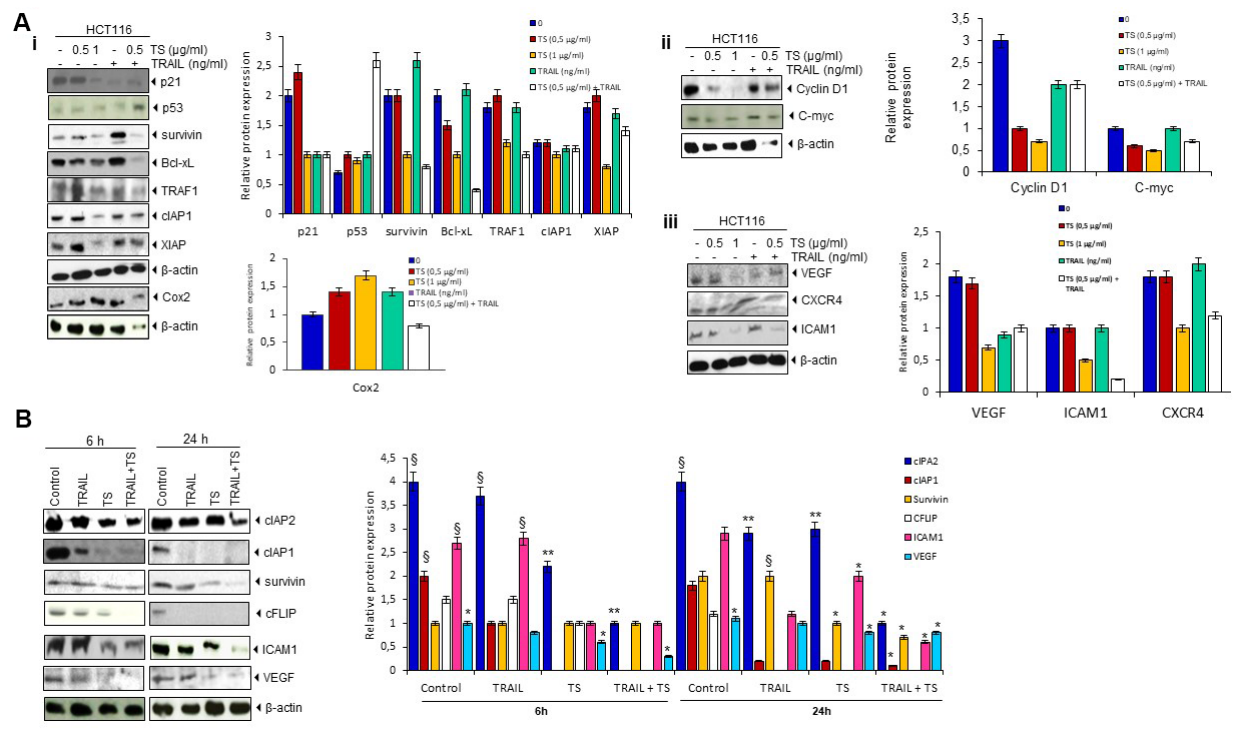
(**A**) TS downregulates (**A**i) antiapoptotic, (**A**ii, **A**iii) proliferative, and metastatic gene products. Briefly, colon cancer cells were treated with indicated stimuli for 24 h. Whole cell protein extracts were prepared, separated by electrophoresis, and then transferred to the nitrocellulose membrane using the antibodies indicated and relative protein expression levels were quantified. (**B**) TS sensitizes TRAIL-induced change in antiapoptotic, proliferative and metastatic gene product levels. HCT116 cells (1 × 10^6^ cells/well) treated with either thyme volatile oil and TRAIL for the indicated times (6 h and 24 h). Whole-cell lysates were subjected to Western blotting analysis using relevant antibodies and relative protein expression levels were quantified. The same blots were stripped and reprobed with β-actin antibodies to verify equal protein loading. These are representative results of three independent experiments. * *P*˂ 0.05, ** *P* ˂ 0.01 compared with control group. § *P* ˂ 0.05 compared with TS group.

The increase of death receptor level after TS treatment correlated with the down-regulation of various gene products (Figure 5A) that mediate inflammation, cell proliferation, cell survival, invasion, and angiogenesis. We observed that treatment of either TS or TRAIL alone decreased the expression of cIAP-1, cIAP-2, survivin, cFLIP, ICAM-1 and VEGF. The effect of these two agents was more in 24 hours exposed cells. We also observed prominent suppression in these proteins when TS combined with TRAIL (Figure 5B).

### TS inhibits LPS-induced inflammation and carcinogenesis in mice

**Figure 6:**
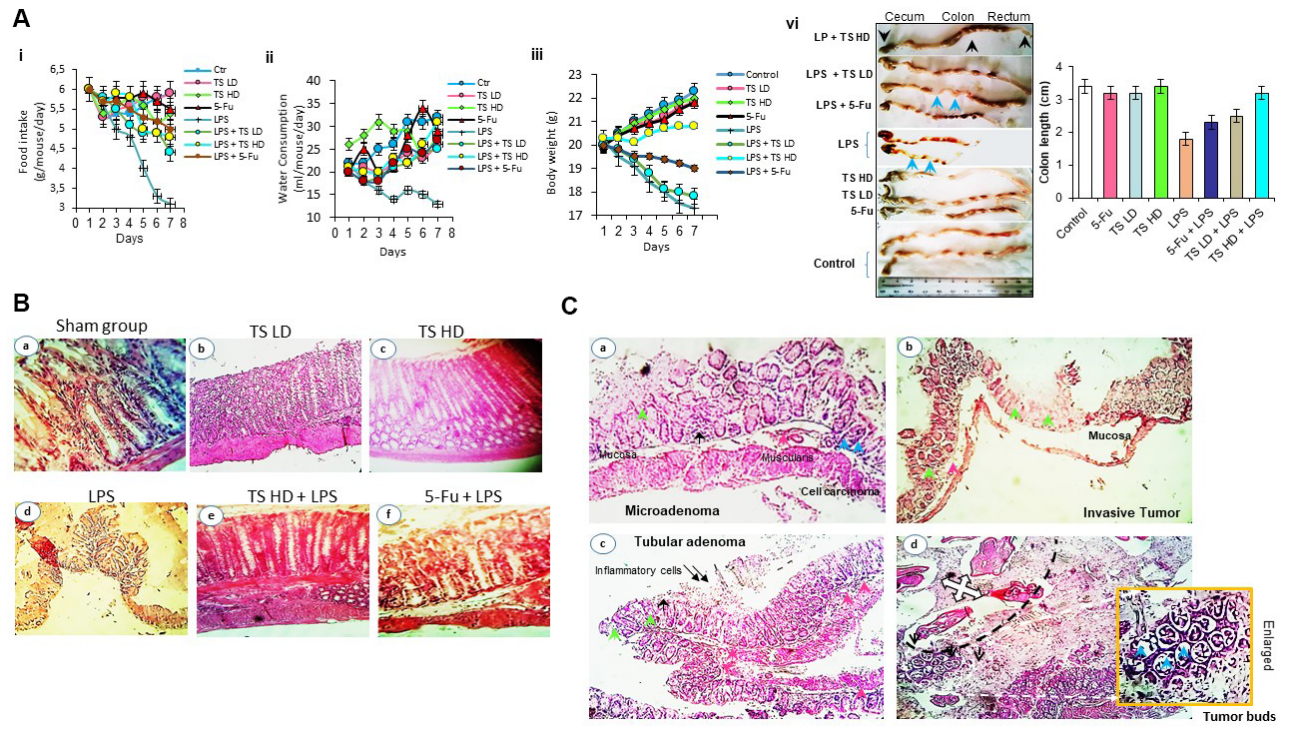
(**A**) Food intake (ii), water consumption (iii), body weight (vi) of mice treated with thyme essential oil (12.5 mg/ml and 50 mg/ml), LPS or both LPS and essential oil and colon length (vi) of different groups treated with drugs. (**B**) Histomorphological evaluation of mouse bearing colon tumor. a-d: normal colon mucosal with intact epithelium; e, f: colon carcinoma; g, h: mild injuries in colon; i: tissues damage (H&E staining, Magnification b, c, e ×10; a, d, f ×40). (**C**) Photomicrograph showing colon colorectal adenocarcinoma in LPS-treated groups. Microadenoma (a); invasive adenocarcinoma (b); tubular adenoma with inflammatory cells (c) and tumor buds (d). Black arrows-inflammatory cell infiltration within mucosa and pink arrows-inflammatory cell infiltration within submucosa; blue arrow-cancer cells, green arrows-crypt and goblet cell loss (H&E staining, Magnification ×40).

Next we determine the effect of TS in animal model. We observed no morbidity, no signs of toxicity and no spontaneous behavioral changes in mice after 7 days of treatment with low dose of TS. These findings suggest safety of TS oil. However, as seen in Figure 6A, body weight loss, lesser food and water consumption were detected in LPS alone and with combination-treated mice during experiments. In contrast, a moderate gain in body weight in TS-treated mice was observed. After 7 days, we sacrificed the mice and analyzed macroscopically. We observed that oral administration of LPS significantly induce damage to colon that demonstrate shortened length (Figure 6Avi). In contrast, TS administration significantly inhibited colonic shortening.

Colon appearance in untreated group (Figure 6Ba), TS (Figure 6Bb, 6Bc) had normal architecture, whereas in the colon tissues from the LPS-induced group were significantly marked by inflammation, lesions and carcinogenesis (Figure 6Bd, 6C) with tumor buds. As shown in Figure 6Cd, the black dashed line separates the tumor mass on the top left from the neoplastic cells or tumor budding on the bottom right. Notably, in group treated with increasing doses of TS, we clearly found reduction in injuries. 5-Fu and LPS-treated mice have induced tissues damage compared with thyme volatile oil treated group.

## DISCUSSION

In Tunisia, ethnobotanical study and biological activity of *Thymus algeriensis* are poorly recorded. EO of *T. hirtus* Ssp. *algeriensis* had a leishmanicidal activity against both Leishmania species with an IC_50_ value (calculated by using regression analysis and expressed as mean 50% leishmanicidal activity) equal to 0.43 μg mL^-1^ for *L. major* and 0.25 μg mL^-1^ for *L. infantum.* Interestingly it was not cytotoxic towards murine macrophagic RAW264.7 (ATCC, TIB-71) cells treated with a concentration of 1 μg/ml after 24 h of incubation [24]. Moreover, lack of cytotoxicity towards macrophagic cells was detected at concentration 8.35 μg/ml for thyme extracts obtained by acetonitrile/water (ACN/W) extraction (60%-40%) and >400 μg/ml for water extract thyme [25]. Thyme essential oil cytotoxicity might be due to its lipophilic compounds that accumulate in cancer cell membranes and increase their permeability, resulting in leakage of enzymes and metabolites [26]. Many EOs and their constituents lead to PARP cleavage [27].

This paper focuses on the cancer preventive and therapeutic potential of thyme essence. We report here that TS inhibit cell viability of various types of tumor cells at different time points. Recently, it has been reported that the antiproliferative effect of oily fractions was correlated with major terpenes constituents, α-Bisabolol as a sesquiterpene alcohol, and (+)-epi-Bicyclosesquiphellandrene [28]. Previous research has demonstrated that terpenic compounds-rich volatile oil from many species prevent tumor progression. Cancer is attributed to uncontrolled proliferation resulting from abnormal activity of different cell cycle proteins. Therefore, cell cycle regulators are becoming attractive targets in cancer therapy [29]. Nutraceuticals have been shown to have potential in cancer prevention for halting cell cycle progression by targeting one or more steps in the cell cycle [30]. The colon cancer regression involve various molecular mechanisms like cell cycle arrest, apoptosis induction as observed by caspase activation and PARP cleavage, antiapoptotic proteins downregulation, p53 induction, angiogenesis, invasion and metastatic gene inhibition. In response to TS treatment, tumor cell proliferation was shown to be suppressed by arresting colon cancer HCT116 cells in the G1 and G2/M phase population through decreasing cyclin D1 expression and upregulating p21 and the tumor suppressor gene p53.

TRAIL receptors have been considered as extraordinary promising antitumor targets, since their cell surface expression preferably kills tumor cells while sparing healthy cells [4]. To gain insight into the antitumor potential of Thyme volatile oil and how it sensitizes TRAIL/Apo2L to induce cell death, western blot analysis was applied. This report shows that TS is a potent chemosensitizer of TRAIL-mediated apoptosis in colon cancer cell lines. Apoptosis was mediated via DR5/DR4 upregulation and downregulation of anti-apoptotic gene expression. TRAIL induces apoptosis by binding to its cognate death receptors (i.e., DR4 or DR5), which then recruit caspase 8 via the FADD. Activated caspase 8 then directly activates caspases-3, -6, and -7 or activates the intrinsic mitochondria-mediated pathway through caspase 8–mediated Bid cleavage, which indirectly activates caspases-3, -6, and -7 [31]. *In vitro* experiments showed that the cotreatment of HCT116 cells with either the highest concentration (0.5 or 1 μg/ml) of TS or TRAIL**/**Apo2L (25 ng/ml) alone significantly enhanced caspase 8 (5-fold), caspase 9 (2-fold) activation and PARP proteolysis (1.5-fold) in dose- and time-dependent manner.

Indeed, thyme is an antitumor agent blocking molecular pathways of colon cancer development. Here, we show that TS significantly induces DR5 expression, compared with that in untreated control cells. Cotreatment of HCT116 cell lines with TS at 0.5 or 1 μg/ml and TRAIL at 25 ng/ml potentiated the upregulation of death receptors. It has been shown that thyme essential oil significantly regulates interferon signaling, N-glycan biosynthesis and ERK5 signaling [26]. Therefore, it has been thought that activated AKT and ERK can function as key effectors of the PI3K and MAPK signaling pathways to promote cell survival and proliferation [32]. ERK1/2 has previously been evidenced to be able of inhibiting the apoptosis effector molecule caspase-3 [33]. Its activation may be crucial in the regulation of Sp1 phosphorylation and consequently Sp1 dependent proapoptotic gene transcription [34]. In this report, higher dose of TS can activate MAPKs signalling pathway, including ERK1/2, c-jun, JNK, p38 MAPK in a dose- and time-dependent manner. Western blot analysis showed that TS mediates the induction of MAPKs at as little as 0.25 and 1 μg/ml concentration in 12 and 24 h of treatment. JNK could be correlated with DR5 expression via Sp1 activation [22]. Therefore, Sp1 can directly bind to the DR5 promoter regions and regulate transcriptional expression [34]. The activation of MAPKs and other kinases (JNK) lead to Sp1 translocation to the nucleus [21]. In this study, we have found that dose-dependent up-regulation of CHOP and the transcription factor Sp1 was detected in TS ethereal oil-treated cells and the highest upregulation was seen at 0.25 μg/ml. Thus, Sp1 and CHOP up-regulation by TS oil involve in death receptor expression. In addition, thyme volatile oil showed HCT116 cell death via JAK/STAT pathway suppression.

**Figure 7:**
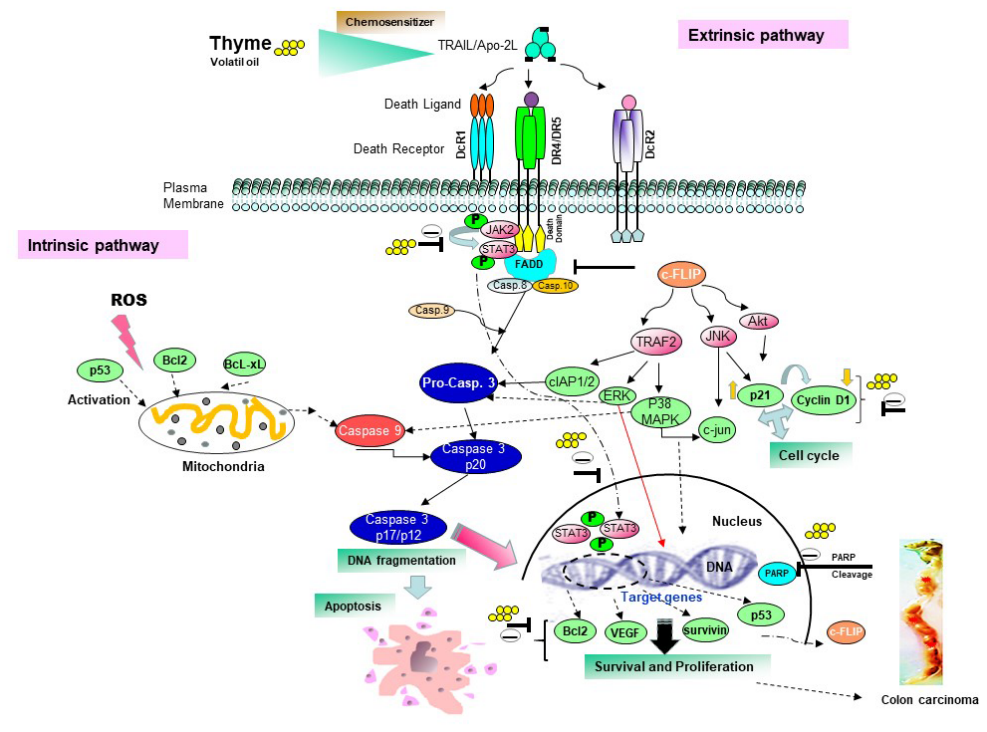
Proposed schematic diagram sensitizes the apoptotic pathway induced by thyme volatile oil and TRAIL/Apo2L on colon cell death.

In this study, we found that exposure of human HCT116 cells to TS sensitized the JAK/STAT and ROS-MAPKs-Sp1/CHOP signal pathway to mediate death receptors expression (Figure 7). TS essence serves as a potent inhibitor of JAK2, which is STAT’s upstream protein kinase [35]. The JAK-STAT pathway plays a pivotal role in transducing signals from the cell surface to target genes in response to cytokines such as IFN-α, IFN-β, IFN-γ, interleukins and growth factors such as EGF, PDGF, GMCSF, and G-CSF [36]. Besides TS oil, several other phytoconstituents belonging to the category of natural terpenoids has shown to inhibit the JAK/STAT pathway by inhibiting the phosphorylation of STAT3 in order to impede cancer cell growth [36].

It is also shown that tumor cells cotreated with TS at 1 μg/ml or TRAIL at 25 ng/ml downregulate the expression of STAT3 target genes (cFLIP, Bcl-xL, ICAM1, VEGF, TRAF1 and the chemokine receptor CXCR4). Chang et al. [37] has shown that c-FLIP_L_, a protease-deficient caspase homolog widely regarded as an apoptosis inhibitor, is enriched in the CD95 death-inducing signalling complex (DISC) and potently promotes procaspase-8 activation through its protease-like domain. Another decreasing expression protein by TS oil was the anti-apoptotic protein Bcl-2. The Bcl-2 family of proteins are key regulators of apoptosis and have been implicated in colorectal cancer (CRC) initiation, progression and resistance to therapy [38]. Combined treatment of cells with TRAIL and thyme essential oil also downregulate anti-apoptotic proteins like survivin, cIAP1/2, XIAP that bind to caspase-3 and -9 and thereby inhibit caspase activity [39]. Transcription factor Sp1 is known to regulate expression of DR5 [40]. In our study, we observed increased expression of Sp1 by TS oil that might participated in induction of apoptosis through upregulation of DR5.

On the viewpoint of cancer events, pharmacological activation of caspase cascades and cleavage of PARP enhances the upregulation of cancer suppressor genes and downregulation of oncogenes, thereby suppressing LPS-induced colon adenocarcinoma. Here again, volatile oil protected mice from LPS-induced colon carcinogenesis and mediated inhibition of inflammation. The protective effect of TS was investigated by the histological examination. We observed antitumor effect of *Thymus* phytocompounds, which was evidenced by decrease in numbers of tumor buds that is a marker of colorectal cancer progression. Notably, tumor budding is defined as single cells or small groups of tumor cells (up to 4 cell clusters) within the tumor or at the invasive front [41]. Tumor buds or small tumor cell clusters have also been closely related to the epithelial-mesenchymal transition [42]. Increasing evidence has shown that epithelial–mesenchymal transition plays a critical role in tumor cell metastasis [30]. In colon cancer patients, high budding correlated with high tumor grade, infiltrating growth pattern, venous invasion, lymphovascular invasion, and infiltration of a free serosal surface [43].

In light of the above facts, TS potentiates TRAIL-induced cell death through death receptors and inhibits growth and proliferation of colon cancer cells. Thus, plant essential oils have the potential application as protective natural drug towards colon carcinogenesis. However, the molecular mechanisms of the volatile oil of *Thymus algeriensis*, its phytopharmacological benefits and safety should be investigated by its application as nutraceutical in clinical trials in human body.

## MATERIALS AND METHODS

### Plant materials

*Thymus algeriensis* plant was collected from the Mountain of Orbata (the southwest of Tunisia) (N 34° 22′ 49.8″ and E 9° 3′ 23.4″). A voucher specimen (n.1188) was deposited in the Herbarium of National Institute of Agronomic of Tunisia (INAT). The terpenic compounds from this plant as shown in Figure 1A was extracted by Clevenger apparatus (50 g dry powdered areal part of the plant in 500 ml distilled water). Obtained essential oil was kept in the dark at -18° C until analysis. Terpenic compounds and polysaccharides of TS were identified by Gas Chromatography and Mass Spectrometry analysis (GC/MS) and FTIR.

### Reagents

Stock solutions of volatile content extracted from TS aerial parts (100 μg/ml) were prepared in dimethyl sulfoxide (DMSO) (Sigma-Aldrich, St. Louis, MO, USA) and were stored at -20° C. TRAIL/Apo2L (TNF-related apoptosis-inducing ligand) was obtained from PeproTech (Rocky Hill, NJ, USA). Sodium dodecyl sulphate (SDS), MTT (3-(4,5-dimethylthiazol-2-yl)-2,5-diphenyltetrazolium bromide), Tris, glycine, LPS (*Escherichia coli* 055:B5), and NaCl were purchased from Sigma-Aldrich Chemicals Co. (St. Louis, MO, USA). BSA (Bovine serum albumin) was obtained from Atlanta Biologicals (Norcross, GA, USA). Roswell Park Memorial Institute medium (RPMI)-640 medium, Iscove’s modified Dulbecco’s medium (IMDM), Dulbecco’s modified Eagle’s medium (DMEM)/F12 medium, streptomycin, Penicillin, and fetal bovine serum (FBS) were purchased from Mediatech, Inc. (Herndon, VA, USA). Antibodies against cIAP1/2 (inhibitor of apoptosis protein), caspases-3, -8, and -9, ICAM1 (Intercellular Adhesion Molecule 1), Bcl-2 (B-cell lymphoma 2), p53, cFLIP (FLICE/caspase-8 inhibitory protein), c-Myc (cellular v-myc myelocytomatosis viral oncogene homolog), p38, Mcl-1 (myeloid cell leukemia), MMP-9 (matrix metalloproteinase), cyclin D1 (cell cycle regulator protein), Bcl-xL (B cell lymphoma extra large), PARP (poly-adenosine diphosphate ribose polymerase), CXCR4 (C-X-C chemokine receptor type 4), STAT-3 (signal transducers and activators of transcription protein), and pSTAT3 (Tyr705 and Ser727) (phosphor-signal transducers and activators of transcription protein), p-ERK1/2 (phosphor-extracellular signal-regulated kinases ½), ERK2 (extracellular signal-regulated kinase 2), p-JNK1 (phosphor-c-jun N-terminal kinases), JNK1 (c-jun N-terminal kinases),, p-Akt (phospho-protein kinase B), Akt (protein kinase B), JAK2 (Janus-activated kinase 2), CHOP (CCAAT/enhancer-binding protein homologous protein), SP1 (specificity proteins 1) and DR4 were purchased from Santa Cruz Biotechnology (Santa Cruz, CA, USA); Anti-DR5 was purchased from ProSci, Inc. (Poway, CA); Antibodies against survivin were purchased from R&D Systems (Minneapolis, MN, USA); Antibody against XIAP (X-chromosome-linked IAP) was obtained from BD Biosciences (San Diego, CA, USA); Antibody against VEGF (vascular endothelial growth factor) was purchased from NeoMarkers (Fremont, CA, USA); Anti-DcR1, anti-DcR2, anti-c-FLIP were obtained from BD Biosciences and Imgenex (San Diego, CA, USA); Dichlorodihydrofluorescein diacetate was purchased from Invitrogen. Antibody against β-actin was obtained from Sigma-Aldrich (St. Louis, MO, USA). 5-FU (5-Fluorouracil) (purity > 99%) was purchased from Sigma Chemical Co.

### Cell lines

The human cancer cell lines HCT116 (colon cancer), KBM-5 (chronic myeloid leukemia cells), Panc28 (pancreatic carcinoma), U266 (multiple myeloma), SCC4 (squamous cell carcinoma), MCF7 (breast adenocarcinoma), and RAW 264.7 (mouse monocyte macrophage) were obtained from the American Type Culture Collection (Manassas, VA, USA). HCT116, SCC4, Panc-28 and RAW cells were cultured in Dulbecco’s modified Eagle’s medium (DMEM) supplemented with 10% FBS (fetal bovine serum), 100 U penicillin and 100 U streptomycin. MCF-7 cells were grown in DMEM/F12 supplemented with 10% FBS. U266 were cultured in RPMI1640 supplemented with 10% FBS, 100 U/ml penicillin and 100 μg/ml streptomycin. KBM-5 cells were cultured in Iscove’s modified Dulbecco’s medium (IMDM) supplemented with 15% FBS and penicillin-streptomycin. Cells were cultured in cell culture flasks and maintained in a humidified incubator (5% CO_2_ at 37° C). Experiments were performed in the exponentially growing cells.

### GC/MS analysis

To obtain the chemical profile of TS terpenic compounds, gas chromatography coupled with mass spectrometry (GC/MS) analysis was conducted on an Agilent 6890 gas chromatograph with an autosampler coupled with an Agilent 5973 Mass Selective Detector (MSD) (Agilent Technologies, Palo Alto, CA, USA) with an electron impact ionization of 70 eV. A Phenomenex ZB- 5MSi capillary column (30 m × 0.25 mm, 0.5 μm film thickness; Agilent Technologies, Hewlett-Packard, CA, USA) was used at a temperature programmed to rise from 40 to 280° C at a rate of 5° C/min using helium (99.999% purity) as a gas carrier, with a flow rate of 0.7 mL/min, a split ratio of 60:1, and a scan time and mass range of 1s and 50–550 *m*/*z*, respectively. EO constituents were identified by matching the mass spectra recorded with those stored in the Wiley 09 NIST 2011 mass spectral library of the GC/MS data system.

### Cytotoxicity assays

The cytotoxic effect of TS oily fractions was assessed by the MTT assay as described earlier [44]. Briefly, HCT116, Panc28, MCF7, SCC4, KBM5, U266 and RAW 264.7 cells (5 × 10^3^ cells per well) were pretreated with increasing concentrations of TS essential oil (0, 0.5, 1, 5, 10, 50 pg/ml) in triplicate onto 96-well plates. The cells were incubated at 37° C for various durations (24, 48, and 72 h). Control wells were incubated with vehicle. Tumor cells were incubated with MTT solution (20 μl/well) for 2 h at 37° C, then extraction buffer (SDS + dimethyl formamide) was added and cells were incubated overnight at 37° C. Absorbance were measured at 570 nm by an MRX Revelation 96-well multiscanner (Dynex Technologies, Chantilly, VA, USA) where extraction buffer was considered as a blank.

### Live/dead assay

To measure TS-induced cell death, we used Live/Dead assay (Esterase Staining Method). HCT116 (2×10^3^) were seeded in 96-well plates and treated with increasing dose of TS essential oil (0.1-5 pg/ml) at 37° C. The medium was removed and washed with PBS and then incubated with live/dead reagent (Calcein AM+PBS+ Ethidium homodimer) for 30 min. Calcein AM, a non fluorescent dye, produce intense green fluorescence when retained within live cells and Ethidium homodimer solution produce red fluorescence when retained by damaged plasma membrane of dead cancer cells. The percentages of cells in apoptosis were calculated by counting live and dead cell numbers. Tumor cells were analyzed under a fluorescence microscope (Labophot-2; Nikon, Melville, NY, USA).

### Colony formation assays

Single tumor cell growth into a colony was investigated through clonogenic assay. The clonogenic assay represents the capacity of a cell to grow after a series of mitosis over a 9-day period [45]. For this, HCT116 cells (5 × 10^2^ cells per well) were seeded into 6-well plates. Following attachment, cells were exposed to different concentrations of diluted TS essential oil (0.01, 0.05, 0.1, 0.5, 1 μg/ml), TRAIL (25 ng/ml), and TS+TRAIL for 24 h. One day later, the medium was replaced with fresh medium, and allowed to form colonies for an additional 9 days. During 9 days of incubation, medium was replaced once with fresh medium. At the end of experiment, colon cancer cells were washed with PBS, fixed with clonogenic acid reagent and incubated for 20 min. After that, colonies were stained with crystal violet dye (0.5%) for 30 min, washed twice, and blue colonies were counted.

### Western blot analysis

Tumor cells were treated with TS and/or TRAIL. After completion of exposure, cells were harvested and incubated with lysis buffer. Lysed cells were centrifuged (1400 rpm g/10 min) at 4° C and then supernatant was collected in fresh Eppendorf tube. Total protein content was measured by BCA method (Uptima, France) using BSA (Serum Bovine Albumin) -as standard. Lysates (30 μg) of treated and untreated cells were resolved in SDS-PAGE (10%) and then proteins transferred onto the nitrocellulose membranes. After being washed with PBS-T, membranes were blocked with nonfat dry milk (5% in PBS-T) for 2 h and then probed with the relevant primary antibodies (overnight/ 4° C): cIAP-1/2 (1:2000), Bcl-xL (1:2000), Bcl-2 (1:3000), ICAM-1 (1:2000), PARP (1:5000), caspase-3 (1:2000), -9 (1:2000) and -8 (1:2000), CXCR4 (1:5000), cyclin D1 (1:3000), c-FLIP (1:3000); VEGF (1:1000), c-Myc (1:2000), MMP-9 (1:1000), p53 (1:3000), Mcl-1 (1:3000), survivin (1:2000); XIAP (1:3000), TRAF1 (1:2000), pSTAT3 (Tyr^705^ and Ser^727^) (1:2000) and β-actin (1:10,000). Membranes were blocked with nonfat dry milk (5% in PBS-T) and then incubated with secondary antibodies, with dilution factor of 1:5000. Blot signals were detected by Enhanced chemiluminescence reagent (GE Healthcare, Piscataway, NJ, USA). The resulting bands were quantitated by NIH imaging software (https://imagej.nih.gov/ij/download.html).

### TS protects mice from LPS-induced colon carcinoma

### Animals

Forty eight Swiss albino mice (20 ± 2 g weight, 5-week-old) were kept in propylene cages with 6 mice/group under cycle of 12 h light/dark at room temperature (18-25° C) and a relative humidity of 55-65%. Animal experiment design was carried out according to Guide for the Care and Use of Laboratory Animals (8th edition, National Academies Press) and approved by the Animal Ethics Committee, University of Carthage at Tunis, Tunisia (No. LNSP/Pro 152012). Animals received standard diet and free access to tap water.

### Study setting

Animals were divided into 8 groups (n=6 mice/group) as follows: (1) saline-treated sham group, (2) LPS (oral 10 μg/ml) treated group, (3) TS essential oil (oral 12.5 mg/ml) treated group, (4) TS essential oil (oral 50 mg/ml) treated group, (5) 5-FU (oral 20 mg/ kg/day) treated group, (6) both TS and LPS (oral 12.5 mg/ml and 10 μg/ml, respectively) treated group (7) both TS and LPS (oral 50 mg/ml and 10 μg/ml, respectively) treated group, (8) both 5-FU and LPS (oral 20 mg/kg and 10 μg/ml, respectively) treated group. Thyme oily fraction was obtained by diluting TS stock solution in the vehicle (2% Tween 80). Intragastric administration of essential oil and comparator drug were given daily 1 h prior to LPS treatment for 1 week. Observations of spontaneous mouse behavior, physical parameters like body weight, water consumption and food intake and clinical responses throughout experiments were recorded daily before the drug dosing. At the end of experiments, mice were sacrificed and organ tissues (colon, liver, heart, spleen, kidney and lung) were immediately collected and processed for macroscopical and microscopical analysis. Colon length of different groups was determined. For histopathological process, organ portions were fixed in buffered formalin solution (10%) and embedded in paraffin wax. Thick sections (5μm) were cut, deparaffinized and stained with hematoxylin and eosin (H&E). Observations were done with Motic SFC-28 SERIES microscopy.

### Statistical analysis

Data were analyzed with mean ± SD. One-way ANOVA followed by a Tukey post hoc test was performed to compare difference between groups.

### Ethics approval

This study was approved by the Research Ethics Committees of the Pasteur Institute, Tunisia (No. LNSP/Pro 152012) in accordance with the Declaration of Helsinki.
